# The additional value of self-reflection and feedback on therapy outcome: a pilot study

**DOI:** 10.3389/fpsyg.2024.1451251

**Published:** 2024-12-23

**Authors:** David Kamp, Cynthia Blanker, Anton Hafkenscheid, Jim van Os

**Affiliations:** ^1^UMC Utrecht Brain Center, Utrecht, Netherlands; ^2^Psychiatrie Rivierenland Tiel, Tiel, Netherlands; ^3^Arkin/Sinai Centre, Amersfoort, Netherlands

**Keywords:** therapeutic alliance, reflective practice, psychotherapy, deliberate practice, process research

## Abstract

**Introduction:**

Over the past few decades, psychotherapy research was dominated by testing the efficacy of “brand name” therapeutic techniques and models. Another line of research however, suggests that common factors, such as the therapeutic alliance and empathy, might play a greater role in effective therapy than specific therapeutic techniques and models. Routine process monitoring (RPM), focusing on common factors, has emerged as a promising approach to enhance therapy outcomes. This ongoing feedback loop aims to improve the therapeutic alliance and to address countertransference issues, potentially leading to better therapy outcomes.

**Methods:**

A total of 131 patients above the age of 18 were included into the sample. The design of the study consisted of three stages, in which two kinds of instruments to measure the therapeutic alliance and countertransference were administered, namely the Session Rating Scale (SRS) and a brief version of Impact Message Inventory-Circumplex (IMI-CS). The Outcome Rating Scale (ORS) was used as an outcome measure. Depending on the stage of the study, these three instruments were administered every time a patient had a psychotherapy session.

**Results:**

Patients reported improved outcomes on the ORS in T2 compared to T1 (*p* = 0.011). Furthermore, a significant increase in the strength of the therapeutic relationship, as measured by the SRS, occurred between T2 and T3 (*p* = 0.031). A positive correlation was found between SRS scores and the “friendly” position on the IMI-CS (*p* = <0.000), while a negative correlation was found between SRS ratings and the “submissive” position on the IMI-CS (*p* = 0.019).

**Discussion:**

Patients experienced improvements and might attribute part of their improvement to the relationship with their therapists, evaluating the therapeutic alliance more positively in the later stages of their treatment. Despite methodological challenges, such as varying treatment durations and pre-existing therapist-patient relationships, the findings highlight the value of patient engagement and therapist self-reflection. This study supports the utility of deliberate practice in psychotherapy, suggesting it can enhance therapeutic outcome and laying the groundwork for future research.

## Introduction

The psychotherapeutic environment should ideally consist of two (or more) self-reflecting individuals, sharing thoughts on a certain issue, in order to develop more insight that can give rise to actual change (Van and Kamp, 2019). The psychotherapist’s role is to support the thought and affectional processes of the individual seeking his help. Rogers (1951) described three core conditions for psychotherapy to be helpful: congruence, empathy and unconditional positive regard. With the rise of cognitive behavioral techniques in the 1980s and 1990s, focus shifted from the therapeutic environment, currently referred to as a “common factor,” to the narrowly defined psychotherapeutic techniques and models used, the so-called specific factors (Wampold, 2010; Cuijpers et al., 2019). Over the past few decades, numerous therapeutic techniques were developed with the intent to improve therapeutic outcomes. Despite all efforts, however, limited progress has been made in terms of psychotherapy efficacy and effectiveness (Van Oenen, 2019). In present day psychotherapy practice, the focus may be shifting back more to where it was when Rogers developed his client-centered therapy. While it is likely that both specific factors (therapeutic techniques) and common factors (i.e., therapeutic alliance, goal consensus, empathy) converge to bring about therapeutic change, no consensus exists on *how much* each of these factors contribute (Mulder et al., 2017). Wampold (2015) makes a strong case that common factors contribute to a greater degree to outcome than specific factors. It could be expected that this would have resulted in a rapid growth in research examining the components and mechanisms of common factors. However, this has barely been the case. The apparent trend is that the search for the best therapeutic techniques and methods should continue, as this has been the primary focus of psychotherapeutic research conducted in the past decades and represents that trend toward “wanting to win” in Psychology (Haeffel, 2022). Small differences between different “brand name” psychotherapies have been reported, but these typically disappear when controlling for therapist allegiance (Dragioti et al., 2015). This poses the question why “novel” psychotherapies continue to be developed, given that all therapies developed to date are about equally effective. Perhaps it does not matter so much *which* particular “bona fide” type of therapeutic method or technique is used, but rather the fact *that* one is used. In addition, what works at group level (which is what is being researched in RCTs), may not work for particular individuals within the group. Thus, results based on group comparisons may not apply to the process of within-person change. Indeed, even the process of assigning patients to a particular diagnostic category is problematic as patients within the same diagnostic category typically differ as much from each other as they do in comparison with patients in other categories (Van Os et al., 2019; Fisher et al., 2018). Thus, given the limited applicability of group-based findings reported in RCTs at the level of individual patients, acknowledging that there are “different strokes for different folks” may be in order (Blatt and Felsen, 1993). What may work for one patient, may not work for another patient.

Research suggests that mobilizing common factor techniques has a positive bidirectional association with the therapeutic alliance (Solomonov et al., 2018). A promising alternative for standardizing treatment interventions into packages’ (protocols) is the implementation of standardized monitoring instruments in unstandardized (tailor-made) psychological treatments. This strategy implies that patients and/or therapists routinely monitor their “inner worlds” on a regular (session-by-session) base: patients routinely reflecting on the impact of each treatment session, therapists routinely reflecting on the impact messages (“silent language”), conveyed by patients’ interpersonal styles and motives. The concept of “impact messages” is grounded in the relational dynamics between patient and therapist, particularly within the framework of interpersonal and psychodynamic theories of psychotherapy (Kiesler, 1996). These messages refer to the implicit emotional signals or “silent language” conveyed by patients, often reflecting their relational patterns, interpersonal motives, and underlying attachment styles (Horowitz, 2004). Such signals can evoke strong emotional responses in therapists, known as countertransference, which includes both conscious and unconscious internal reactions to the patient. Recognizing and interpreting these messages is crucial for therapists to maintain therapeutic neutrality and adapt their interventions effectively. The model supporting this approach draws heavily from interpersonal theory (Sullivan, 1953) and psychodynamic concepts of transference and countertransference (Freud, 1912, 1915; Kernberg, 1984). Routine process monitoring (RPM) operationalizes this relational feedback loop by encouraging therapists to systematically reflect on these emotionally charged reactions as part of the therapeutic process. Using validated monitoring tools, RPM provides a structured avenue for both parties to explore and address these dynamics, fostering greater awareness and a more tailored therapeutic alliance. This process bridges the gap between subjective relational experiences and evidence-based practices, enhancing the efficacy of psychotherapy by integrating real-time emotional insights into the treatment framework.

Such reflections are systematized by having patients and therapists complete standardized and validated monitor tools. This process is also known as Routine process monitoring (RPM), as opposed to Routine outcome monitoring (ROM), where the main focus is on outcomes rather than the dynamics of the psychotherapy. Routine process monitoring encourages patients to be aware of positive and negative experiences in the “here and now” of treatment sessions. At the same time, therapists enhance their own awareness of countertransference reactions by tracking their own feelings regarding patients’ in-treatment behaviors. When a therapist experiences strong emotionally charged negative cognitions, feelings and action tendencies (impact messages) toward a particular patient, his treatment is likely to be less effective or may even fail. This highlights the importance of being aware of one’s countertransference in psychotherapy.

The Session Rating Scale (SRS; Duncan et al., 2003; Hafkenscheid et al., 2010; Janse et al., 2017) is a simple, well-validated monitor instrument, intended to capture patients’ session evaluations in only four items. The Outcome Rating Scale (ORS; Miller et al., 2003; Hafkenscheid et al., 2010) is a 4-item questionnaire, routinely tracking (positive, negative or lack of) changes in the patients’ everyday life over the course of treatment. Whereas the SRS and ORS focus on the patient’s perspective, the Impact Message Inventory-Circumplex (IMI-C) focuses on the pleasant and unpleasant internal reactions patients elicit in their therapists. The Impact Message Inventory-Circumplex (IMI-C; Hafkenscheid, 2018) is an extensively researched monitor tool for therapists, dedicated to explore their feelings about patients’ interpersonal communication (“command messages” or “impact messages”) during the therapeutic encounter. The IMI-C consists of 56 items. However, a psychometrically validated brief 32-items IMI-C has been proposed by Sodano et al. (2013): the IMI-CS (Short). This paper investigates the use and impact of both standardized self-reflection tools (SRS for patients and IMI-CS for therapists) on intermediate treatment outcomes, as measured with the ORS.

The current study was performed in an outpatient mental health care facility in The Netherlands, Psychiatrie Rivierenland Tiel (PRT). PRT treats a heterogeneous group of patients varying from mood disorders to personality disorders and from psychotic syndromes to autism spectrum disorder. The vast majority of patients treated at PRT are linked to both a psychiatrist and a psychologist. The aim of the study was to conduct a pilot to test the influence of routine process monitoring in psychotherapy. We aimed to pilot the sequential use of SRS, ORS and IMI-CS with different levels of active use of these during ongoing psychotherapy in a sample of patients undergoing psychotherapy in a routine clinical setting. Hypothesis 1: by monitoring the overall wellbeing of the patient (ORS) and his/her satisfaction of the therapeutic alliance (SRS) on a session by session base enhances overall wellbeing (ORS). Hypothesis 2: when therapists assess their own countertransference after each session (IMI-CS), this would positively affect overall wellbeing (ORS). Hypothesis 3: when patients evaluate their overall wellbeing (ORS) and satisfaction with the therapeutic alliance (SRS) after each session, combined with the therapist assessing countertransference (IMI-CS) after every session, this would improve overall wellbeing (ORS).

## Materials and methods

### Participants

The sample consisted of 131 participants who were recruited for the study at the Rivierenland mental health care facility. Both patients already receiving treatment at the facility and patients commencing treatment at the start of the experiment were recruited. Only patients younger than 18 years were excluded from participation. No other exclusion criteria were applied.

### Procedure

Over the course of their treatment, patients were sequentially exposed to three stages (T1, T2, and T3). Each stage lasted 2 months. Thus, each patient participated for 6 months in the study. T1 we denote as the Patient Only Self-reflection/No feedback-condition, T2 as the Patient and Therapist Self-reflection/No feedback-condition, T3 as the Patient and Therapist Self-reflection/Feedback-condition.

*Patient Only Self-reflection/No feedback-condition*: In T1, patients filled out both SRS and ORS at the end of each session. SRS and ORS were not shown to their therapists.

**Figure 1:**
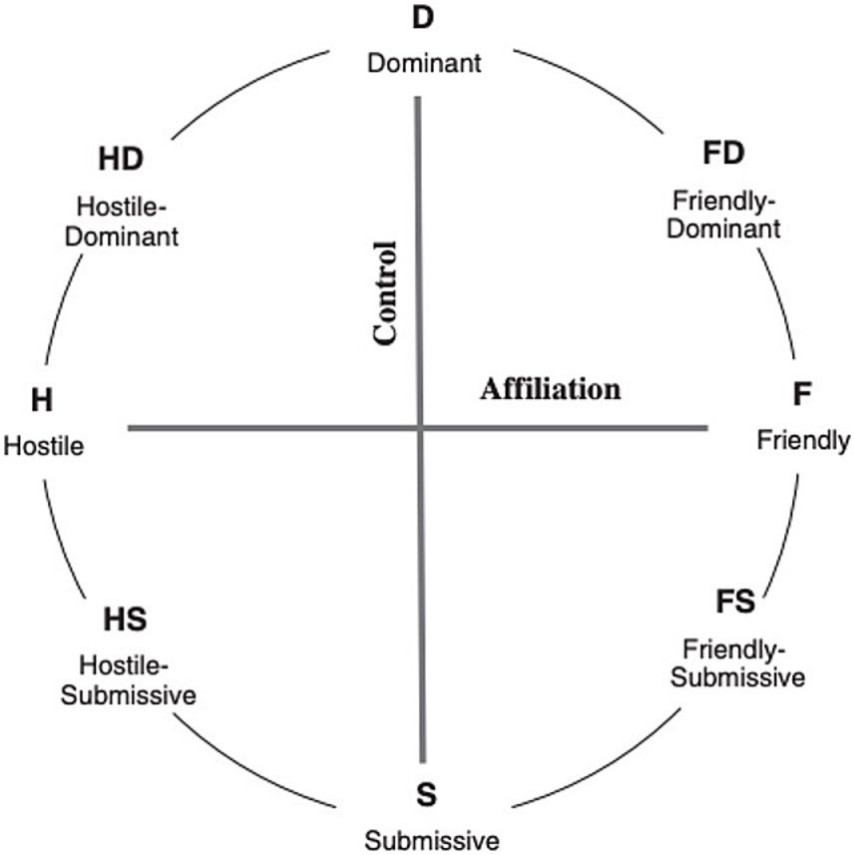
The interpersonal circle (Kiesler, 1983; Wiggins, 1979) shown with octant subscale labels of the impact message inventory–circumplex (Kiesler and Schmidt, 2006). Permission acquired by Hafkenscheid.

*Patient and Therapist Self-reflection/No feedback-condition*: In T2, patients continued to fill out the SRS and ORS. In addition, therapists filled out the Dutch version of the shortened Impact Message Inventory Circumplex (IMI-CS) after each session. Filling out the IMI-CS produced a figure called the Interpersonal Circle model (IPC; Kiesler, 1983; Wiggins, 1979) as shown in Figure 1. Patients were blinded to the IMI-CS ratings of their therapists.

*Patient and Therapist Self-reflection/Feedback-condition*: In T3, patients continued to fill out the ORS and ORS on a session-by-session basis, whereas the therapist did the same for the IMI-CS. Different from the second stage, ORS and SRS ratings were unblinded to the therapist. Moreover, ORS and SRS ratings were discussed in dialog between patient and therapist. This resulted in 5–10-min conversations between therapist and patients, evaluating the previous session, but also “overall” psychotherapeutic processes. Common questions a therapist would ask the patient were: “You scored a 6 (SRS) on ‘approach or method’, what do you think is needed to get to a 7?” or “On ‘relationship’ you scored a 10 (SRS), what makes the relationship between us so good in your point of view?.” Therapists were given the option to discuss the impact messages with their patients as well, but this was left to the personal preferences of the therapists. Patients were not ignorant of the therapist filling out the IMI-CS, however.

In each 2-month period, patients received a maximum of 10 sessions, or 1 weekly session at most. The number of sessions varied between 0 and 10 for each condition. The total number of sessions varied between 1 and 19, given a possible maximum of 30 sessions.

### Instruments

The instruments used were the Session Rating Scale (SRS), Outcome Rating Scale (ORS) and the Dutch version of the brief Impact Message Inventory Circumplex (IMI-C Short).

The Session Rating Scale (SRS) consists of 4 items inquiring about the current therapy session: relationship, goals and topics, approach and method, overall. The following items were scored on a 100 mm line: relationship, goals and topic, approach or method and overall (Duncan et al., 2003). The patient was asked to mark a hash mark on the line. A hash mark depicted on the left side of the line represents negative responses whereas positive responses are depicted with a hash mark on the right side of the line. The higher the rating, the more positive the self-reported evaluation. One SRS sample item: “Relationship” was evaluated with the item “I did not feel heard, understood, and respected” to “I felt heard, understood, and respected.”

The Outcome Rating Scale (ORS) also consists of 4 items. These items enquire about how the patient was feeling over the past week. The following items are scored on a 100 mm line: individually (personal wellbeing), interpersonally (family and close relationships), socially (work, school, friendships), Overall (general sense of wellbeing) (Duncan et al., 2003). The patient is asked to mark a hash mark on the line. A hash mark depicted on the left side of the line represents negative responses whereas positive responses are depicted with a hash mark on the right side of the line.

The Dutch version of the brief Impact Message Inventory Circumplex (IMI-C Short) consists of 32 items (Sodano et al., 2013). IMI-C Short scores are graphically depicted in the interpersonal circle (IPC). The IPC is divided into the following eight octants: dominant, friendly-dominant, friendly, friendly-submissive, submissive, hostile-submissive, hostile, hostile-dominant, as shown below in Figure 1.

The purpose of this instrument is to help the therapist understand his countertransference reactions to their patients, also called “impact messages” (Hafkenscheid, 2012). Each of the first 20 items of the IMI-CS starts with the phrase *“When I am with this person, (s)he makes me feel ….”* The last 12 items start with the phrase *“When I am with this person, it appears that… “.*A sample item from the dominant octant scale *“… bossed around.”* The items are rated on a 4-point Likert scale, with 1 denoting “Not at all” and 4 denoting “Very much so.” The eight octant scores are connected by continuous line showing the absolute and relative position of patients’ pattern of impact messages in the interpersonal circle, as experienced by the therapist.

### Analysis

Data analysis was performed using STATA 16 (2019). Data was analyzed in the long format, each patient contributing multiple observations, each observation representing a session, with a maximum of 30 sessions (three conditions with a maximum of 10 sessions each). Multilevel random intercepts regression models, taking into account hierarchical clustering of observations within patients, were fitted with SRS and ORS as dependent variables and “condition” as independent variable, *a priori* adjusting for age and sex. In order to assess the influence of IMI-C Short on SRS and ORS outcomes, the means of the IMI-C Short on the positions dominant, submissive, friendly and hostile were added as additional covariates.

## Results

### Demographics

A convenience sample of 131 patients participated in the study. The number of months patients received therapy at the current facility prior to the start of the data collection varied from 0 to 72 (*MEAN* = 29, *SD* = 24). The number of months of therapy received during the research period varied between 0.6 and 6 months (*MEAN* = 5.7, *SD* = 1). The age of the sample varied from 18 to 82 years with a median of 49. Of the 131 participants, 81 were women and 50 were men. Sample demographics and details are provided in Table 1.

**Table 1:** Summary of participants.

	*N* (%)	Age (MEAN/SD)	Treatment duration at PRT in months (MEAN/SD)
Male	50 (38%)	51/15	28/24
Female	81 (62%)	46/15	30/25
Total	131	48/15	29/24

N, Number of individuals; SD, Standard deviation; PRT, Psychiatrie Rivierenland Tiel (research site).

During T2, the IMI-C Short was administered 217 times across 98 patients. The IMI-C Short was administered 113 times across 59 patients during T3.

### Regression analyses

#### Association of ORS, SRS, and IMI-C short with condition over time

Hypothesis 1 predicted that patients would score higher on the ORS during their continuing participation in this study, across the three conditions. There was a positive adjusted association between ORS and condition, in that compared to T1, ORS was higher in T2 (B = 4.853, *p* = 0.011) and also in T3 (B = 5.474, *p* = 0.006), with no significant difference between T2 and T3 (*p* = 0.778). This indicates that most of the total improvement of wellbeing occurred in T2 and persisted in T3 (Table 2).

**Table 2:** Results of ORS and SRS across the 3 phases of the experiment.

	*N*	MEAN	SD	Std. error mean
ORS T1	119	23	8.7	0.8
ORS T2	71	24	9.3	1.1
ORS T3	61	24	9.8	1.3
SRS T1	119	33.7	5.3	0.49
SRS T2	70	33.2	6.4	0.76
SRS T3	62	34.2	5.1	0.66

ORS, Outcome rating scale; SRS, Session rating scale, SD, standard deviation.

Analyzing the SRS across the three experimental conditions over time revealed no differences in the degree to which patients were positive about the therapy between T1 and either T2 (B = -1.000, *p* = 0.463) or T3 (B = 2.403, *p* = 0.093). There was, however, a significant increase in the degree to which patients were positive about the therapy between T2 and T3 (*p* = 0.031).

Hypothesis 2 predicted that when therapists assess their own countertransference after each session (IMI-CS), this would positively affect overall wellbeing (ORS). No large or significant association was found between the scores on the ORS and the IMI-CS however. The aforementioned results apply to T2 and T3, given that the IMI-CS was not administered during T1.

Hypothesis 3 predicted that when patients evaluate their overall wellbeing (ORS) and satisfaction with the therapeutic alliance (SRS) after each session, combined with the therapist assessing countertransference (IMI-CS) after every session, this would improve overall wellbeing (ORS). In T2 and T3, the average perceived quality of the therapeutic relationship, as assessed by the SRS, was positively associated with the “friendly” position within the interpersonal circle, as measured by the IMI-CS (*p* = <0.000). Additionally, a negative association was observed between the average perceived quality of the therapeutic relationship and the “submissive” position within the interpersonal circle (*p* = 0.019). No large or significant association was found between the scores on the ORS and the IMI-CS however. The aforementioned results apply to T2 and T3, given that the IMI-CS was not administered during T1.

### Association of ORS and SRS with each other over time

In the model of ORS, there was a significant association with scores on the SRS (B = 0.225, *p* = 0.001) with no evidence of difference of this association between the three conditions (T1, T2, T3) over time (*p* interaction = 0.213). Similarly, in the model of SRS, there was a significant association with ORS (B = 0.120, *p* = <0.000), with no evidence for interaction with the three conditions (*p* = 0.676).

## Discussion

This pilot study suggests that deliberate practice may improve efficiency and efficacy of clinical practice: patients reported improvement over the course of study and attributed part of their recovery to their therapist and the relationship they had established with their therapist. During the last 10 weeks of the experiment, patients evaluated the therapeutic alliance more positively.

Of course, the findings need to be interpreted in the light of several methodological limitations. Only several patients started their *treatment* simultaneously with the *start of the data collection*. Most were already in treatment before data collection commenced. For future research we recommend replication studies to start simultaneously with the first treatment session. This way, a more direct link between the treatment process and treatment outcome can be made. Also, the evaluation of the therapeutic alliance may provide more room for change when patients start off with a new therapist at the start of the study and growth in the alliance is more likely to be observed. The majority of patients in the current study had already known their therapist for a relatively long time, up to 6 years in one case. Nonetheless, patients improved during the experiment, whether they had been in treatment for years or only for some months.

Data were incomplete as not all sessions were rated as required, mostly for logistical reasons. Additionally, the duration of therapy treatments among patients varied widely. The advantage of this variation is that the study’s results are applicable to a broad spectrum of patients, ranging from those receiving their first-ever treatment to those who have been undergoing therapy for up to 6 years, as observed in this study. This variation more accurately reflects everyday clinical practices. However, a significant limitation was that in many cases, a therapeutic alliance had already been established, and special attention to patient feedback prior to this study was often lacking. We hypothesize that dedicating more attention to patient feedback throughout the treatment process from the first to the final session could foster a stronger therapeutic alliance (Budesa, 2020).

This study differed from treatment as usual in that patients had been given more autonomy in their own therapy. This could very well have been a change in their treatment compared to their treatment before the study commenced. Furthermore, therapists were encouraged to actively reflect on the therapy, the patient, themselves and the complex interaction of these three components.

Patients expressing greater satisfaction with therapy in the later stages of the experiment could be attributed to significant improvements in their wellbeing earlier in the process. This positive feedback may reflect a sense of gratitude toward the therapist. However, prior research often observed a contrary phenomenon, where a strong therapeutic alliance was reported before any notable improvements in wellbeing were evident (Martin et al., 2000). The therapist rated Impact Message Inventory Circumplex (IMI-CS), an instrument utilized for monitoring therapist countertransference, was administered from the second period of the study onward. A positive association was identified between the “friendly” position on the IMI-CS and the therapeutic relationship, as measured by the Session Rating Scale (SRS). This suggests that when therapists perceive patients as being engaged in collaboration, patients concurrently experience the therapeutic relationship as more positive. Of course IMI-CS responses are an inevitable interaction between countertransference reactions in the original definition as well as accurately describing the patients’ interpersonal style. This constitutes a hypothesis-confirming outcome. Additionally, during the third phase of the study, we found a negative association between therapists perceiving patients as “submissive” and patients’ perceptions of the therapeutic relationship. This suggests that patients view the quality of the therapeutic relationship less positively when their therapists feel they occupy a more passive, “submissive” role. According to the interpersonal circle theory, this dynamic may also lead therapists to adopt a complementary “dominant” stance. We strongly support the integration of routine process monitoring in psychotherapy, akin to deliberate practice, and see it as a key component. Deliberate practice, a well-documented catalyst for success across diverse fields like music, sports, business, and psychotherapy, emphasizes the need for focused and intentional improvement efforts (Ericsson and Pool, 2016; Rousmaniere, 2016). The growing fascination with deliberate practice is well-founded, especially as psychotherapy has seen little advancement in patient outcomes over recent decades. Identifying the most impactful factors has become crucial, as the development of new therapeutic methods has not led to significant progress. Deliberate practice, however, holds promise for the evolution of psychotherapy (Miller et al., 2015). Given the results of this pilot study, our research team is currently embarking on a larger-scale study to delve deeper into how deliberate practice influences psychotherapy, with a refined research design to capture its effects more accurately.

In conclusion, this pilot study underscores the potential benefits of implementing self-reflection and feedback mechanisms into psychotherapy, according to the principles d of deliberate practice. Through the structured use of client-rated instruments the Session Rating Scale (SRS), Outcome Rating Scale (ORS), and therapist-rated instrument the Impact Message Inventory-Circumplex Short (IMI-CS), this research offers a glimpse into the positive shifts in therapy outcomes that such practices can engender. While it is true that the outcome rating scale (ORS) and session rating scale (SRS) were initially designed for clinical use to facilitate feedback and improve therapeutic outcomes, their utility in research settings has become increasingly evident. These tools are brief, user-friendly, and adaptable to diverse clinical environments, making them ideal for examining therapy processes in real-time. The ORS and SRS capture essential aspects of the therapeutic relationship and patient outcomes with minimal administrative burden, enabling high compliance and consistent data collection, even in busy clinical settings. Moreover, the psychometric properties of the ORS and SRS have been rigorously validated, demonstrating reliability and sensitivity to change, which are crucial for research purposes (Duncan et al., 2003; Campbell and Hemsley, 2009; Hafkenscheid et al., 2010). Their ability to detect subtle shifts in the therapeutic alliance and patient wellbeing aligns with the objectives of psychotherapy research, where understanding process dynamics is key. In this study, the ORS and SRS were particularly valuable in providing a continuous, session-by-session account of therapeutic progress, offering insights into the interplay between alliance quality, patient engagement, and treatment outcomes.

Furthermore, their application in our study aligns with the principles of routine process monitoring (RPM), where the primary focus is on improving therapeutic processes rather than solely on endpoint outcomes. By incorporating these measures into a research framework, we were able to bridge the gap between clinical practice and scientific inquiry, demonstrating how practical tools can enhance the understanding of therapeutic mechanisms while maintaining their relevance in everyday treatment contexts. Participants in this study reported an improvement in their wellbeing over time, which correlated positively with the therapeutic alliance fostered with their therapists. Perhaps participants attribute their progress to their well-established relationship with their therapist. We did not ask them this directly however. Notably, a more favorable evaluation of this alliance was observed in the latter stages of the study, suggesting that the interventions implemented may have contributed to strengthening these relationships. It is unlikely that development over time explains these improvements, since a vast portion of the sample had already been in therapy for a significant period of time. However, it is important to recognize the limitations of this study, such as the pre-existing therapist-patient relationships and logistical challenges that led to incomplete data. Despite these hurdles, the findings point to the value of enhancing patient engagement and therapist self-reflection in the therapeutic process. The positive association between the “friendly” position in the IMI-CS and patient perceptions of the therapeutic relationship, coupled with the negative view associated with perceived “submissiveness,” highlights the complex dynamics at play in therapy sessions. These insights underscore the importance of therapist awareness and adaptability in fostering a conducive therapeutic environment.

This study advocates for a broader adoption of routine process monitoring in psychotherapy, resonating with the principles of deliberate practice known to drive success across various domains. The promising results from this pilot study pave the way for further research on a larger scale, aiming to delve deeper into the mechanisms through which deliberate practice can enhance psychotherapeutic outcomes. The continued exploration of these practices holds the potential to refine and evolve psychotherapy, making it a more effective and responsive tool for addressing mental health needs.

## Data Availability

The raw data supporting the conclusions of this article will be made available by the authors, without undue reservation.
